# Construction of a Real-Time Detection for Floating Plastics in a Stream Using Video Cameras and Deep Learning

**DOI:** 10.3390/s25072225

**Published:** 2025-04-01

**Authors:** Hankyu Lee, Seohyun Byeon, Jin Hwi Kim, Jae-Ki Shin, Yongeun Park

**Affiliations:** 1 Department of Civil and Environmental Engineering, Konkuk University-Seoul, 120, Neungdong-ro, Gwangjin-gu, Seoul 05029, Republic of Koreashinjaeki@gmail.com (J.-K.S.); 2 Division for Integrated Water Management, Korea Environment Institute, Sejong 30147, Republic of Korea; shbyeon1@gmail.com; 3 Future and Fusion Lab of Architectural, Civil and Environmental Engineering, Korea University, Seoul 02841, Republic of Korea; 4 Limnoecological Science Research Institute Korea, THE HANGANG, Gyeongnam 50440, Republic of Korea

**Keywords:** plastic debris monitoring, deep learning, object detection, YOLO, water management

## Abstract

Rivers act as natural conduits for the transport of plastic debris from terrestrial sources to marine environments. Accurately quantifying plastic debris in surface waters is essential for comprehensive environmental impact assessments. However, research on the detection of plastic debris in surface waters remains limited, particularly regarding real-time monitoring in natural environments following heavy rainfall events. This study aims to develop a real-time visual recognition model for floating plastic debris detection using deep learning with multi-class classification. A YOLOv8 algorithm was trained using field video data to automatically detect and count four types of floating plastic debris such as common plastics, plastic bottles, plastic film and vinyl, and fragmented plastics. Among the various YOLOv8 algorithms, YOLOv8-nano was selected to evaluate its practical applicability in real-time detection and portability. The results showed that the trained YOLOv8 model achieved an overall F1-score of 0.982 in the validation step and 0.980 in the testing step. Detection performance yielded mAP scores of 0.992 (IoU = 0.5) and 0.714 (IoU = 0.5:0.05:0.95). These findings demonstrate the model’s robust classification and detection capabilities, underscoring its potential for assessing plastic debris discharge and informing effective management strategies. Tracking and counting performance in an unknown video was limited, with only 6 of 32 observed debris items detected at the counting line. Improving tracking labels and refining data collection are recommended to enhance precision for applications in freshwater pollution monitoring.

## 1. Introduction

Plastic debris causes severe pollution in freshwater environments and is a major contributor to ocean pollution. As plastic debris from freshwater systems flows into rivers and lakes, it eventually reaches the sea, adversely affecting aquatic ecosystems. Over time, plastic debris decomposes due to various environmental processes. Photodegradation, driven by ultraviolet (UV) rays from sunlight, and physical abrasion from waves, wind, and biological interactions break down plastics into smaller fragments [1,2]. These processes generate microplastics, which are small enough to be easily ingested by aquatic organisms. Microplastics accumulate within the tissues of these organisms, leading to bioaccumulation. This process intensifies the concentration of microplastics and associated toxic chemicals as they ascend the food chain to higher predators. Such bioaccumulation can negatively affect top predators, such as fish, by impairing their reproductive capacity, growth, and survival [3]. Moreover, when humans consume these contaminated organisms, potential health risks arise [4]. In response to these challenges, global initiatives to mitigate plastic pollution in freshwater environments have been mobilized in line with the United Nations 2030 Agenda for Sustainable Development. SDG 6 (Clean Water and Sanitation), SDG 11 (Sustainable Cities and Communities), and SDG 14 (Life Below Water) are identified as critical targets for addressing these environmental issues [5].

Pollution caused by plastic debris in freshwater environments has resulted in numerous documented cases of ecological and environmental harm. Research has identified injuries to fish and birds in freshwater ecosystems due to plastic debris, either through direct contact or the ingestion of plastics fragments mistaken for food [6]. Such ingestion can inflict damage on internal organs and hinder nutrient absorption, thereby threatening species survival [7]. Furthermore, reports indicate that otters in the Mississippi River [8] have suffered entanglement in plastic debris, leading to severe injury or mortality. These instances highlight the detrimental consequences of plastic pollution on freshwater ecosystems. Historically, research on plastic waste in freshwater environments has predominantly focused on the collection and analysis of plastic debris from rivers and lakes. For instance, Cable et al. [9] conducted extensive investigations in the Great Lakes region of North America, collecting plastic pollutants and examining their distribution and movement. Their findings showed that the highest concentrations of floating plastic were observed in urban areas with high population density, reinforcing the role of rivers and streams as primary pathways for transporting plastic waste to marine environments. Similarly, Blettler and Mitchell [10] analyzed plastic waste retrieved from sediment deposits in freshwater lakes, categorizing the debris by size. Their study determined that the majority of macroplastics entering freshwater systems originated from household sources, with food packaging, beverage bottles, and plastic bags identified as the principal contributors. They further cautioned that macroplastics could degrade into microplastics over time, posing a threat to freshwater ecosystems and potentially to human health.

Research by Cable et al. [9] and Blettler and Mitchell [10] has demonstrated that the direct collection of plastic waste in freshwater environments provides critical insights into the types and sizes of plastic pollutants, including microplastics. However, this method has significant limitations. While it enables detailed analysis of contamination, it is highly labor-intensive and may pose safety risks, particularly when accessing remote or hazardous study sites. Consequently, traditional methods may not always be viable for long-term or large-scale monitoring efforts. Advancements in computer and satellite technology have addressed the labor-intensive nature of conventional plastic-collection methods, offering safer and more efficient alternatives. Kikaki et al. [11] successfully detected plastic waste in the Bay of Bengal using data from Landsat-8 and Sentinel-2 satellites, as well as high-resolution imagery from Planet Labs. By analyzing the spectral characteristics of plastics and distinguishing them from seaweed, they successfully detected 20 instances of plastic debris. Similarly, Basu et al. [12], employed Sentinel-2 satellite multispectral images to classify floating plastic debris in marine environments. Their study identified that visible blue, green, red, near-infrared, and infrared spectral bands were effective in detecting floating plastic, achieving detection accuracy between 96.9% and 98.4% through the application of supervised learning algorithms.

The emphasis on large lakes, river estuaries, and coastal regions has yielded valuable insights into the dynamics of plastic accumulation and potential removal strategies. However, the primary limitation of these studies lies in their scale, making it difficult to directly apply their findings to freshwater river systems. In such systems, plastic transport and accumulation dynamics may differ due to variations in flow rates, narrower channels, and distinct geographical features. As a result, there is a critical need for targeted research on smaller freshwater systems to develop more localized and effective strategies for mitigating plastic pollution in rivers. To address these limitations, recent studies have utilized unmanned aerial vehicles (UAVs) equipped with cameras to enhance plastic waste detection. Cortesi et al. [13] employed a UAV equipped with a multispectral camera to detect plastic waste discharged into rivers. Using a random forest-based classifier, the researchers successfully distinguished plastic from other materials. While they achieved 100% detection accuracy at an altitude of 30 m, the precision was notably low, at only 3.1%. UAV-based research presents a significant advantage by enabling the remote detection and classification of floating plastic debris in freshwater environments. However, this approach requires specialized UAV operation skills and is challenging to implement under windy conditions [14].

The advancement of object-detection algorithms using deep learning has markedly improved the accuracy and efficiency of automated object detection. Lieshout et al. [15] implemented a deep learning-based monitoring system by installing cameras on bridges over rivers in Jakarta to differentiate macroplastic debris from other environmental elements. Their automated approach achieved a detection accuracy of 68.7% and identified approximately 35% more plastic debris compared to manual visual counting conducted by researchers. While this method effectively detects the presence of plastic, its primary limitation lies in its inability to classify different types of plastic waste, providing only binary data on plastic presence.

Accordingly, this study aims to develop a deep learning-based automatic monitoring algorithm for detecting floating plastic debris using a systematic process that includes field video data collection, manual annotation, and the application of a YOLOv8-nano detection framework. The proposed system performs object detection, classification, and quantification of plastic debris, thereby enabling real-time monitoring of riverine plastic pollution. A critical question addressed in this study is whether different types of plastic can be accurately detected in scenarios where heavy rainfall upstream results in a substantial influx of plastic into the river. Moreover, this study emphasizes evaluating the model’s practical applicability under field conditions. Ultimately, by tackling this challenge, the research seeks to provide a more precise and cost-effective alternative to traditional monitoring methods.

## 2. Materials and Methods

### 2.1. Data Acquisition

**Figure 1:**
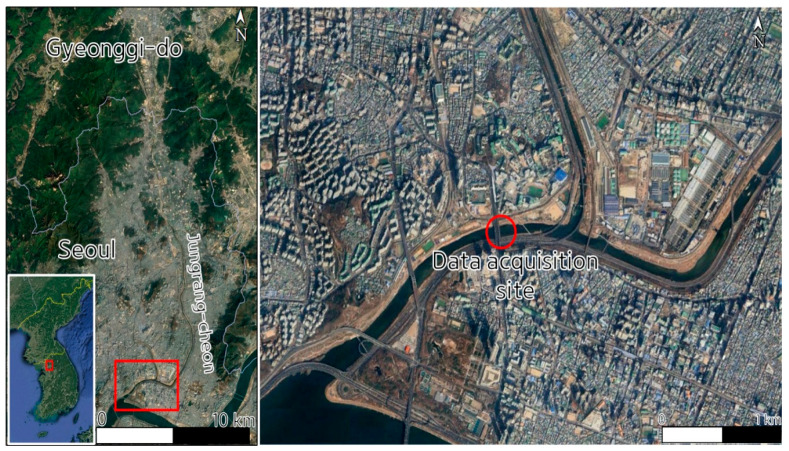
Details of the acquisition location for the floating plastic debris image samples used in the dataset construction (GPS Coordinate: 37°33′09″ N, 127°02′38″ E).

The video of floating plastic debris was collected downstream of Jungrang-cheon, from the bridge closest to the Han River (Figure 1). This location was strategically selected to facilitate effective monitoring of plastic waste transported into the Han River, providing essential data for analyzing the movement of plastic debris from urban areas into the river system. The recording took place on 29 June 2023, two hours after a heavy rainfall had passed through the upstream region of Jungrang-cheon, located in North Gyeonggi Province, South Korea. Detailed information on temporal rainfall events in the data acquisition area is provided in Table S1. The video was captured using an action camcorder (GoPro9Hero, GoPro, Inc., San Mateo, CA, USA) mounted on the bridge, positioned at a 15-degree angle relative to the water surface and oriented upstream (Figure S1). The video resolution was set to 1920 × 1080 pixels and saved in the Moving Picture Experts Group-4 Part.14 (MP4) format. The video was recorded at 30 frames per second, with a total length of 90 min.

### 2.2. Data Preprocessing

The Computer Vision Annotation Tool (CVAT; Boris et al. [16]) was deployed in a local environment to annotate the recorded video. Bounding boxes were meticulously drawn around each instance of floating plastic debris on the water surface to ensure precise coverage, facilitating accurate detection and classification. The bounding box information was saved in a text (“txt”) file format, adhering to the annotation input requirement of the You Only Look Once (YOLO) series. Macroplastic debris identified in the video was classified through visual inspection into four categories such as common plastics (plastic_normal), plastic bottles (plastic_bottle), plastic film and vinyl (plastic_film), and other fragmented plastics (plastic_fragments). Each observed macroplastic object was annotated in every video frame. To maintain consistency between the annotations and the video data, all frames containing annotated objects were extracted, ensuring a one-to-one correspondence between the image frames and annotation files. Consequently, the dataset comprised a total of 4162 images with corresponding TXT annotation files, along with 1005 background class data entries. For model training, validation, and testing, all input images were randomly divided in a 7:2:1 ratio, respectively.

### 2.3. Detecting, Tracking, and Counting Algorithms

#### 2.3.1. Modified YOLOv8

**Figure 2:**
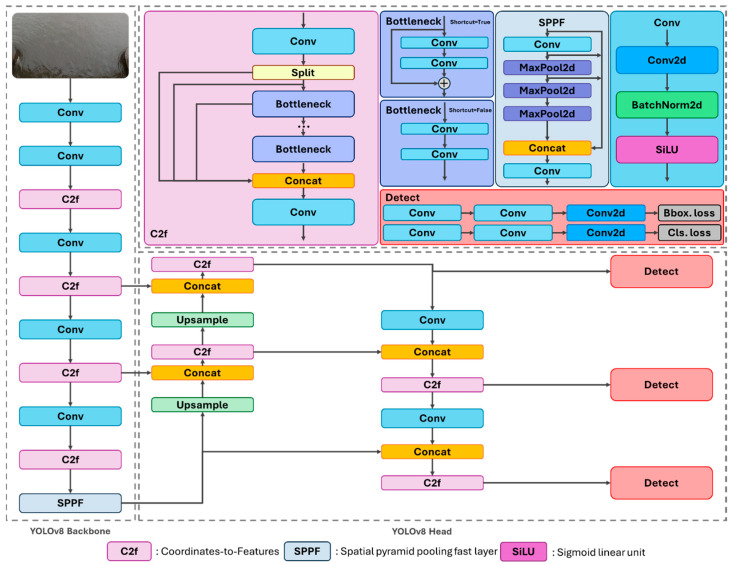
YOLOv8 network architecture: detailing backbone and detection layers.

The automatic plastic debris monitoring model was developed using the You Only Look Once (YOLO) version 8 algorithm (Figure 2) [17], specifically designed for the efficient detection of floating plastic debris on the water surface. YOLOv8, a state-of-the-art one-stage object-detection algorithm, has demonstrated superior performance, achieving lower inference latency and higher mean average precision (mAP) performance on the COCO dataset [18]. YOLOv8 supports various pre-trained weights, such as YOLOv8n (nano), YOLOv8s (small), YOLOv8m (medium), YOLOv8l (large), and YOLOv8x (extra-large), allowing researchers to select an appropriate model based on computational resource and study requirements [19]. In this study, the smallest pre-trained model, YOLOv8n, was chosen due to its portability and computational efficiency. YOLOv8n is particularly well-suited for edge deployment and real-time applications, as it offers a compact architecture, faster inference speed, and lower computational demand compared to larger YOLO models, such as YOLOv8l or YOLOv8x [20]. The backbone architecture of YOLOv8n was retained without modifications, and the model was trained after adjusting the hyperparameters to align with the research objectives. Details of the modified hyperparameters and system configuration are provided in Table 1. The backbone architecture of YOLOv8n was retained without modifications, and the model was trained after adjusting the hyperparameters to align with the research objectives. Details of the modified hyperparameters and system configuration are provided in Table 1. The image size was set to the maximum supported by YOLOv8 to facilitate more effective feature extraction, given that the objects in the training images are very small. Moreover, the learning rate was determined to be 0.01, which is the optimal value recommended when using the SGD optimizer [21,22].

#### 2.3.2. Deep-SORT

**Figure 3:**
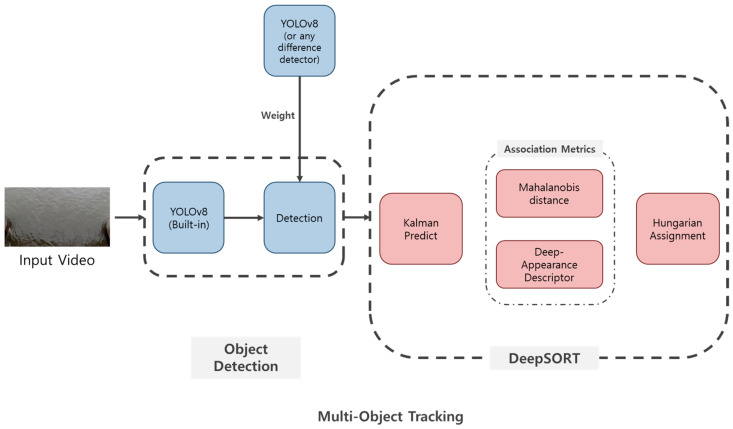
Simplified flow chart of Multi-Object Tracking. In this diagram, any well-known object detection model, such as R-CNN or YOLO, may be employed for object detection. For a detailed workflow of the DeepSORT algorithm, please refer to Supplementary Material Figure S1.

In this study, the Deep-SORT algorithm was utilized to efficiently aggregate object-tracking data. Deep-SORT is an enhanced version of the traditional SORT algorithm, combining a Kalman filter and the Hungarian algorithm for optimal object tracking, along with Mahalanobis distance and a Deep Appearance Descriptor to significantly improve tracking accuracy (Figure 3) [23].

The Kalman filter updates the predicted location of an object based on its motion, while the Hungarian algorithm performs optimal matching between detected objects and predicted positions. The Mahalanobis distance is employed to calculate how close a newly observed object is to a previously tracked one, based on the object’s state vector *x* and covariance matrix *S*, as defined by the following equation [23]:

(1) $$d_{motion}(i,j)={\left(x_{i}-x_{j}\right)}^{T}S^{-1}(x_{i}-x_{j})$$

Additionally, the Deep Appearance Descriptor learns the visual features of objects and compares them based on visual similarity. The similarity between the appearance vectors f of two objects, extracted using a CNN, is computed using cosine distance, which is represented as follows [23]:

(2) $$d_{appearance}\left(i,j\right)=1-\frac{f_{i}\cdot f_{j}}{\left\|f_{i}\right\|\left\|f_{j}\right\|}$$

Here, *f_i_* and *f_j_* are the appearance vectors of object *i* and object *j*, respectively. The cosine distance measures the similarity between the directions of the two vectors, with values closer to 0 indicating higher similarity.

Finally, the motion and appearance information are combined using a weighted sum to improve tracking accuracy [23]:

(3) $$d_{total}(i,j)={\lambda d}_{motion}(i,j)+{\left(1-\lambda \right)d}_{appearance}(i,j)$$

Here, *λ* is a hyperparameter that controls the relative importance of motion and appearance information. This allows Deep-SORT to maintain object identities with high accuracy, even in occlusion or crossing scenarios, enabling efficient Multi-Object Tracking. In this study, the characteristics of Deep-SORT were leveraged to accurately track the movement of plastic debris and aggregate the trajectories of each object. The detailed flow chart of Deep-SORT was represented in Supplementary Material Figure S1.

#### 2.3.3. Model Evaluation

The performance evaluation of the automated floating plastic-monitoring model developed in this study was conducted based on three key criteria. First, the model’s accuracy in classifying objects was assessed to determine its ability to correctly categorize different types of plastic debris. Second, its precision in detecting the location of objects was analyzed to evaluate the accuracy of bounding box placements. Third, the model’s capability in quantifying the number of objects was examined to ensure reliable counting of floating plastic debris.

##### Object-Classification Performance

The classification performance of the model was evaluated using a confusion Matrix, which assesses the model’s accuracy in classifying objects by considering four outcomes: True Positive (TP), True Negative (TN), False Positive (FP), and False Negative (FN). From these outcomes, key performance metrics such as Precision, Recall, and F1-score were calculated to quantify the model’s classification accuracy [24,25].

##### Object-Detection Performance

The detection performance of the model was evaluated using mean average precision (mAP) [26]. The model’s detection accuracy was measured by calculating the mAP with an Intersection over Union (IoU) threshold of 0.5 (mAP@IoU = 0.5). The IoU threshold of 0.5 is a widely accepted evaluation criterion in pre-trained object-detection models based on Pascal VOC or COCO [26,27]. To assess performance changes at higher thresholds, overall detection performance was evaluated using the average metric over a range of IoU thresholds from 0.5 to 0.95 with intervals of 0.05 (mAP@IoU = 0.5:0.05:0.95) [27].

##### Object-Counting Performance

The model’s counting accuracy was evaluated by comparing the number of objects predicted by the model with the actual number of objects observed manually. This ratio of predicted to actual objects provided a measure of the model’s accuracy in object counting and offered insights into its potential applicability in real-world scenarios.

## 3. Results and Discussion

The training of the automatic floating plastic-monitoring model was terminated early at the 739th epoch, out of a total of 1000 epochs. The model achieved its highest performance at the 639th epoch, and the corresponding weights were saved as the optimal model under the filename ‘best.pt’. Validation and testing of the model were performed using these optimal weights, which were applied to images that had not been used during the training process.

### 3.1. Object Classification

**Figure 4:**
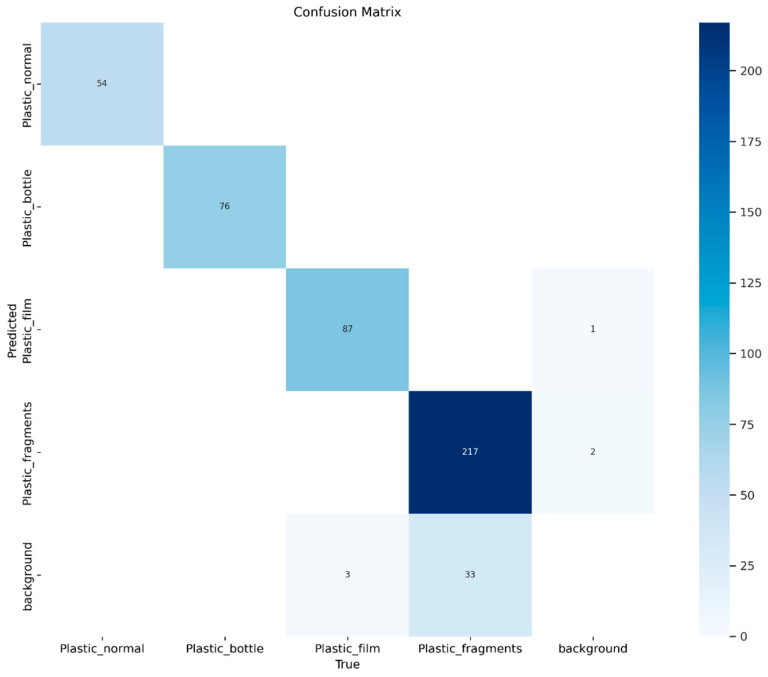
Confusion matrix of model classification result.

**Figure 5:**
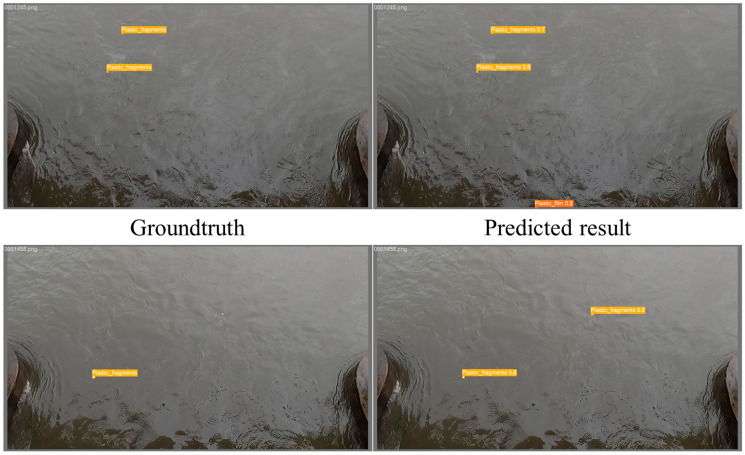
Examples of misclassification: the **upper panel** shows a false positive in the plastic_film class and the **lower panel** in the plastic_fragment class. Although these regions were not annotated in the ground truth, field observations confirmed the presence of plastic film and plastic fragments, demonstrating that the model accurately detected debris omitted during manual annotation.

The classification results of the automatic floating plastic-monitoring model are presented in the confusion matrix (Figure 4). Out of 470 total instances, the model correctly classified 434 instances, achieving an overall accuracy of approximately 92.34%. The classification errors included 36 False Negatives (FN) and 3 False Positives (FP). The false negatives were predominantly observed in the film and fragments classes. Specifically, in the film class, 3 out of 87 objects were not classified, while in the fragments class, 33 out of 217 objects were missed. All false positives occurred in the background class, where 1 film object and 2 fragments objects were misclassified. Several potential factors may have contributed to these classification errors. First, class imbalance may have influenced the model’s performance. The confusion matrix shows that false negatives were concentrated in the film and fragments classes, with the fragment class containing more objects than the other classes. This likely caused the model to overfit to the fragments class, while underfitting the remaining classes, leading to confusion during validation. Visual similarity between classes could also have contributed to the misclassifications. For example, some objects in the film class may have visually resembled those in the fragments class, making it difficult for the model to distinguish between the two. Furthermore, object size might have impacted the classification performance. Objects in the fragments class were generally smaller than those in other classes, and small objects are inherently more challenging to detect and classify, which likely led to higher misclassification rates. For the False Positives (Figure 5), these regions were excluded during the manual classification and annotation stage because they were deemed not to represent objects. However, upon review of the model’s detection results, it was determined that the detected regions corresponded to plastic debris observed in the field. This indicates that the model did not confuse background with objects; rather, it correctly identified objects that were inadvertently omitted during manual annotation. Although such cases may lower performance metrics during classification evaluation, they demonstrate that the model operates as intended in real-world conditions.

The precision, recall, and F1-score for each stage and class are summarized in Table 2. The overall F1-score was 0.982 in the validation stage and 0.980 in the test stage. For the validation stage, the class-wise F1-scores were 0.996 for normal plastics, 0.997 for bottles, 0.993 for film, and 0.941 for fragments. In the test stage, the F1-scores were 0.991 for normal plastics, 0.994 for bottles, 0.985 for film, and 0.947 for fragments. Given that the F1-scores for both the overall model and each class were close to 1.0, the classification performance of the model developed in this study can be considered highly satisfactory.

### 3.2. Object Detection

**Figure 6:**
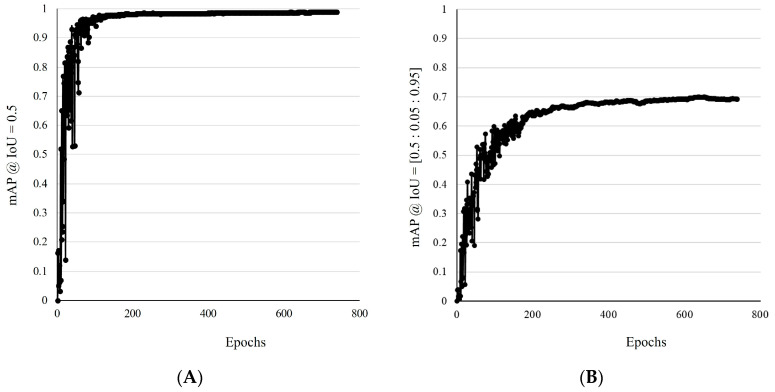
Variation in mean average precision (mAP) at 0.5 IoU (**A**) and 0.5:0.05:0.95 IoU (**B**) across epochs during the validation step. mAP at 0.5 IoU refers to the mAP calculated at a fixed IoU threshold of 0.5 and mAP at 0.5:0.05:0.95. IoU mAP refers to average mAP over multiple IoU thresholds ranging from 0.5 to 0.95, with increments of 0.05.

The detection performance of the automatic floating plastic-monitoring model was evaluated based on two key metrics: mAP at IoU 0.5 and mAP at IoU 0.5:0.05:0.95. In the mAP@IoU = [0.5] matric, the overall model achieved a mAP of 0.990 (Figure 6A), demonstrating highly accurate detection performance during the validation step. Class-wise mAP values were similarly high, with normal plastics (0.995), bottles (0.995), film (0.995), and fragments (0.973) classes all exhibiting excellent detection accuracy (Table 3). During the test step, the model’s overall mAP was slightly higher at 0.992, with individual class mAP values of normal plastics (0.9959), bottles (0.995), film (0.994), and fragments (0.985) confirming the model’s robust performance across different datasets (Table 3). For the more stringent mAP@IoU = [0.5:0.05:0.95] metric, the model’s overall mAP during the validation step was 0.699 (Figure 6B). Class-wise values showed high performance for normal plastics (0.738), bottles (0.736), film (0.720), and fragments (0.601), though a decline in performance was observed, particularly for the fragments class, which had the lowest mAP. In the test step, the overall mAP was 0.714, showing a slight improvement compared to the validation step. Class-wise mAP values were normal plastics (0.754), bottles (0.752), film (0.720), and fragments (0.628), indicating consistent performance across both steps but with persistent challenges in detecting the fragments class. The results indicate that the model performs exceptionally well under the 0.5 IoU threshold, with all classes achieving near-perfect mAP values. This suggests that the model is highly effective at detecting and classifying floating plastic debris when the IoU threshold is lenient. However, the performance decreases significantly when evaluated under the stricter mAP@IoU = [0.5:0.05:0.95] metric, particularly for the fragments class. This decline could be attributed to the small size and irregular shape of the fragments, which makes them more difficult to detect and classify accurately. Additionally, class imbalance may also contribute to this issue, as fragments represent a larger portion of the dataset, potentially leading to overfitting during training and compromising detection accuracy for smaller objects.

The performance of the present study was compared with that of a related previous study [15]. In that work, plastic objects were classified into only two categories (plastic and non-plastic), and the highest mean precision achieved—using horizontal and vertical flip augmentation—was 0.687. Although the IoU criterion used in that study was not specified, even when evaluated using an IoU threshold of 0.5:0.05:0.95, the results of the present study (0.699) indicate superior performance. Thus, the multi-class object-detection model in this study demonstrates excellent performance. It should be noted, however, that the employed dataset was collected from a single location using continuous video recordings at 30 frames per second, which may have induced a natural augmentation effect, potentially contributing to the high performance observed. Future research should incorporate preprocessing techniques, such as an adaptive frame-selection algorithm [28], to minimize uncertainty caused by unnecessary augmentation effects. This approach would help reduce frame-to-frame correlations and enhance the representativeness of the dataset derived from continuous video capture. Also, to address the lower mAP for the fragments class, future work could focus on implementing data augmentation techniques to balance the dataset and improve the detection of small and irregularly shaped objects. Furthermore, with additional training, the model may be extended to detect other types of floating debris, such as non-plastic materials (e.g., wood and metal).

### 3.3. Object Counting

**Figure 7:**
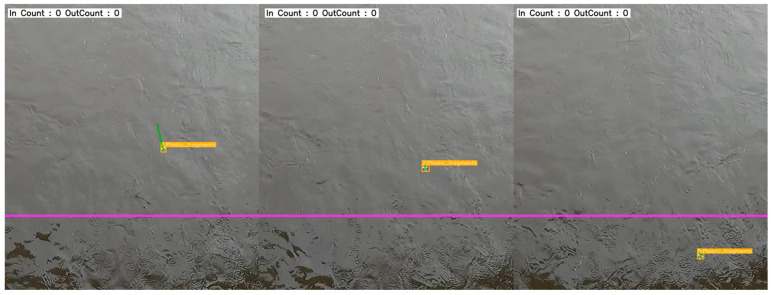
Example of object counting failure. The magenta-colored horizontal line represents the counting boundary used to quantify the plastic debris in the water. In this figure, IDs 1, 2, and 5 represent the same floating plastic debris (class name: plastic_fragments). Initially detected as ID 1, the object temporarily submerged, causing an interruption in its detection and tracking. Upon resurfacing, it was re-identified as ID 2; however, it submerged once more, disrupting tracking before it could cross the counting line. After crossing the line without detection, the object resurfaced and was tracked again, this time assigned as ID 5.

The object-counting algorithm was applied to newly acquired video footage that had not been included in the training, validation, or testing steps. A counting line was defined between the coordinates (1920, 800) and (0, 800), and objects crossing this line during tracking were counted. Out of 32 manually observed pieces of plastic debris, only 6 objects (18.75%) successfully crossed the counting line. Tracking was interrupted for the majority of objects, primarily due to their intermittent submersion and reemergence from the water surface. This behavior frequently led to tracking failures (Figure 7).

Observations indicate that the most pronounced cause of these tracking issues can be attributed to the meteorological conditions during data acquisition. Rainfall led to elevated water levels and increased flow speeds, creating highly dynamic hydrodynamic conditions. Such environmental characteristics can influence the trajectories of floating plastic debris in rivers, and this effect is especially pronounced for smaller, lighter plastic particles [10].

To address the issue of disrupted plastic tracking under dynamic water conditions, future research will strengthen the dataset by incorporating tracking labels and retrain the model to enhance tracking performance. The model’s tracking performance could subsequently be evaluated using Multi-Object Tracking Accuracy (MOTA) and Multi-Object Tracking Precision (MOTP) metrics. Additionally, the Higher Order Tracking Accuracy (HOTA) method proposed by Luiten et al. [29], could be employed for a more comprehensive assessment, as it accounts for both detection accuracy and object consistency, which are crucial for ensuring reliability in complex tracking environments.

An alternative approach for improving performance would involve adjusting the camera angle, which is currently mounted on the bridge, to ensure it is positioned perpendicularly to the water surface. Furthermore, data collection with more selective criteria, particularly for smaller and less distinguishable object classes, could help mitigate performance degradation caused by class similarity.

Furthermore, based on the findings of this study, future research should focus on assembling datasets under diverse meteorological conditions—including periods of rainfall and high solar exposure—to facilitate the development of a generalized model that remains robust across varying weather conditions.

## 4. Conclusions

This study outlines the development and assessment of an automated model for monitoring floating plastic debris, designed to detect and classify plastic waste in aquatic environments. The multi-class detection model, based on the YOLOv8 framework, was trained using field video data captured during a natural rainfall event in a stream. The model accurately classified 434 out of 470 instances across four distinct categories of plastic debris, achieving an overall accuracy of 92.34%. The following key conclusions emerged from this investigation.

- The model’s object-detection performance yielded a mean average precision (mAP) of 0.990 at an Intersection over Union (IoU) threshold of 0.5, and 0.714 under the stricter evaluation range of IoU = 0.5:0.05:0.95. Compared to binary-class detection models from prior studies, this multi-class model demonstrated robust classification and detection capabilities, despite the increased complexity introduced by diverse plastic debris types. These results underscore its potential for practical application under field conditions, particularly during rainfall events and across varying debris morphologies.
- Several factors influencing model performance were identified. Visual similarities between film and fragment debris types posed challenges to accurate detection, contributing to elevated error rates. Additionally, class imbalance—most notably the predominance of the fragments class—may have led to overfitting in that category, while underfitting occurred in others, resulting in misclassifications. To enhance model performance, strategies such as balancing class representation through selective data augmentation and improving feature extraction for small, morphologically similar plastic debris were proposed.
- The tracking and counting of plastic debris proved challenging due to objects frequently submerging and re-emerging under dynamic hydrological conditions. Of 32 tracked debris items, only six were successfully monitored across a designated counting line. This suggests that further improvements are required, particularly in addressing environmental variability, object size, and debris type, to enhance tracking efficacy.

Overall, this study presents a promising methodology for developing an automated classification and detection system for monitoring plastic debris in water bodies. Nevertheless, refinements are necessary to address data imbalance and improve debris quantification. Future research should focus on expanding the dataset by incorporating additional samples collected under diverse hydrological and meteorological conditions. Extending the model’s capabilities to detect other floating debris types, such as wood and metal, would further broaden its practical utility and problem-solving potential. Collectively, these findings advance the field of environmental monitoring technologies and offer valuable insights for water quality management and the mitigation of plastic pollution.

## Figures and Tables

**Table 1:** The details of the modified hyperparameters and the construction environment.

Hyperparameters		Construction Environment	
Image size	1280	GPU	NVIDIA A100 40Gb
Epochs	1000	OS	Ubuntu 22.04
Optimizer	SGD	Software environments	Python 3.12
Learning rate	0.01		Pytorch 2.4.0
Augmentation	90 degrees		Torchvision 0.19.0
	0.5 scale		CUDA 12.1
			CUDNN 9.1.0

**Table 2:** Comparison of classification performance with respect to different types of plastic debris.

	Train/Validation			Test		
	Precision	Recall	F1-Score	Precision	Recall	F1-Score
Overall	0.984	0.980	0.982	0.980	0.979	0.980
Plastic_normal	0.992	1.000	0.996	0.983	1.000	0.991
Plastic_bottle	0.994	1.000	0.997	0.988	1.000	0.993
Plastic_film	0.997	0.989	0.993	0.989	0.982	0.985
Plastic_fragments	0.952	0.930	0.941	0.962	0.932	0.947

**Table 3:** Comparison of detection performance based on mAP for different types of plastic debris.

	Train/Validation mAP		Test mAP	
	IoU = 0.5	IoU = [0.5:0.05:0.95]	IoU = 0.5	IoU = [0.5:0.05:0.95]
Overall	0.990	0.699	0.992	0.714
Plastic_normal	0.995	0.738	0.995	0.754
Plastic_bottle	0.995	0.736	0.995	0.752
Plastic_film	0.995	0.720	0.994	0.720
Plastic_fragments	0.973	0.601	0.985	0.628

## Data Availability

The raw data supporting the conclusions of this article will be made available by the authors on request.

## Supplementary Materials

The following supporting information can be downloaded at: https://www.mdpi.com/article/10.3390/s25072225/s1, Section S1: Meteorological Conditions During Data Acquisition, Figure S1: Installation of the camera at the data collection site, positioned with its lens directed downward toward the water surface to capture optimal images of the floating debris, Figure S2: Field images captured using the camera for four types of floating plastic debris, which were used to construct the model, Figure S3: Detailed flow chart of Multi-Object Tracking.
