# Supplementary material for: Construction of a Real-Time Detection for Floating Plastics in a Stream Using Video Cameras and Deep Learning
Source: Sensors (Basel). 2025 Apr 1;25(7):2225. doi: 10.3390/s25072225 (PMC11991434; doi:10.3390/s25072225)
Supplement: Supplementary file 1 [file sensors-25-02225-s001.zip › sensors-3480312-supplementary.pdf]

Supplementary Materials

S1. Meteorological Conditions During Data Acquisition

The Video was recorded for two hours on June 29, 2023. Rainfall began at 8:00 AM with a depth of 1.5 mm over one hour. There were relatively heavy rainfall events between 11:00 AM and 01:00 PM with a total depth of 37.8 mm over two hours. There was a depth of 13.2 mm during data collection period. Table S1 summarizes the temporal rainfall depths for 16 hours on the sampling day.

Table S1. Hourly rainfall depths from 7:00 AM to 11:00 PM on June 29, 2023 in the study site.

Time	Hour rainfall depth (mm)	
7:00	0	
8:00	1.5	
9:00	2.4	
10:00	3.5	
11:00	14	
12:00	23.8	
13:00	0.9	
14:00	2.2	
15:00	3.2	Data collection period
16:00	9.8	
17:00	0.2	
18:00	0.2	
19:00	1	
20:00	1	
21:00	0.1	
22:00	0	
23:00	0	

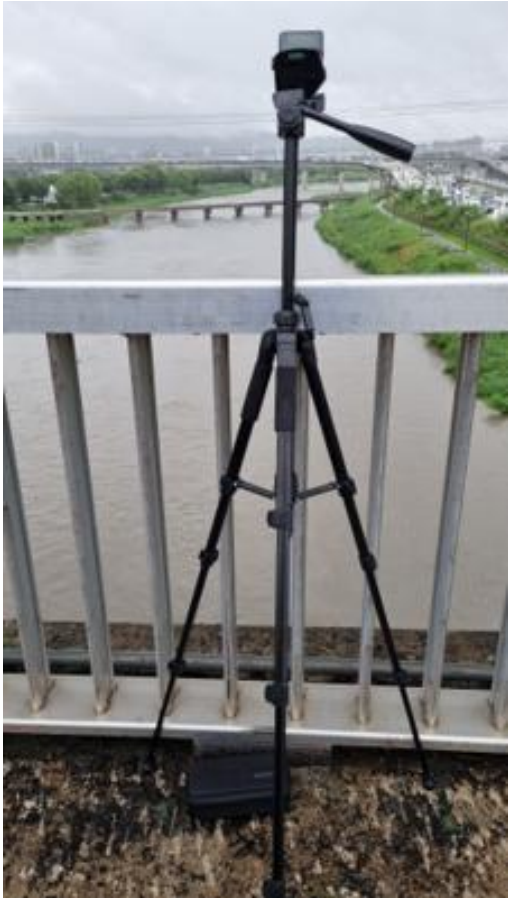

Figure S1. Installation of the camera at the data collection site, positioned with its lens directed downward toward the water surface to capture optimal images of the floating debris.

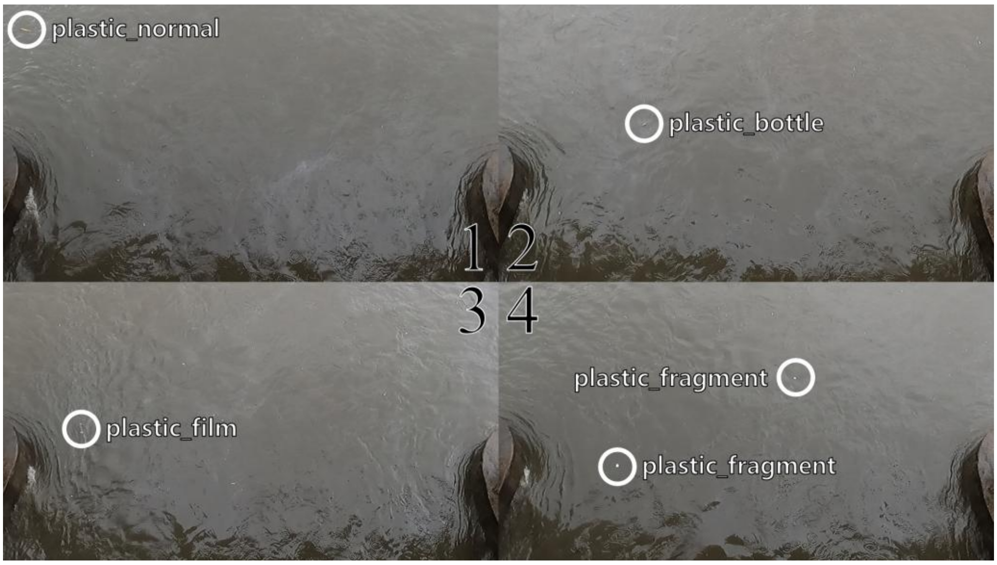

Figure S2. Field images captured using the camera for four types of floating plastic debris, which were used to construct the model.

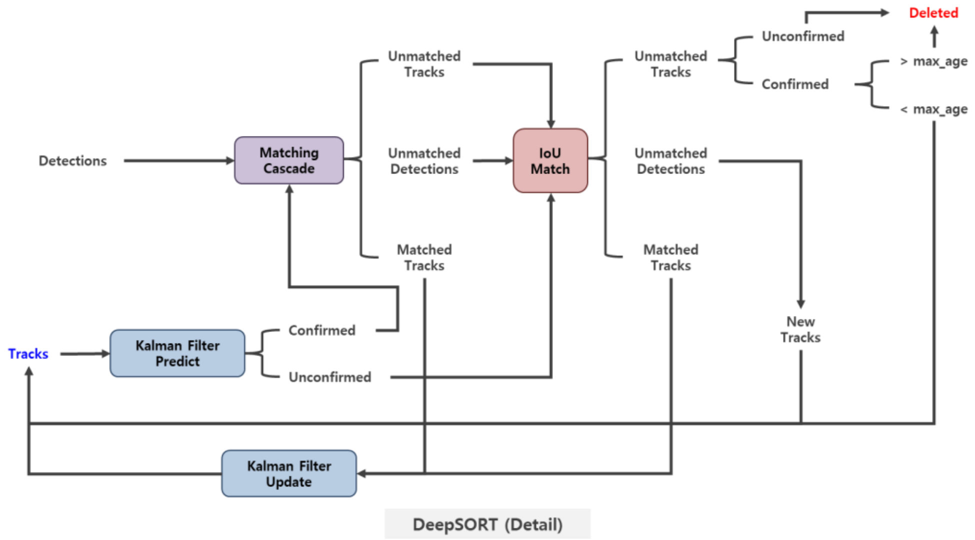

Figure S3. Detailed flow chart of Multi-Object Tracking
